# Effects of Slope and Strip-Cutting Width on Bamboo Shoot Emergence, Culm Formation, and Understory Vegetation Diversity in Moso Bamboo Forests in China

**DOI:** 10.3390/plants15020258

**Published:** 2026-01-14

**Authors:** Dawei Fu, Fengying Guan, Zhen Li, Minkai Li, Yifan Lu, Xiao Zhou, Xuan Zhang

**Affiliations:** 1 International Center for Bamboo and Rattan, Key Laboratory of National Forestry and Grassland Administration/Beijing for Bamboo & Rattan Science and Technology, Beijing 100102, China; fudawei@icbr.ac.cn (D.F.); lizhen@icbr.ac.cn (Z.L.); 13877418610@163.com (M.L.); 17395922296@163.com (Y.L.); zhouxiao@icbr.ac.cn (X.Z.); zhangxuan@icbr.ac.cn (X.Z.); 2 National Location Observation and Research Station of the Bamboo Forest Ecosystem in Yixing, National Forestry and Grassland Administration, Yixing 214200, China

**Keywords:** moso bamboo forest, strip-cutting, understory vegetation diversity, principal component analysis

## Abstract

Moso bamboo (*Phyllostachys edulis*) harvesting is labor-intensive and inefficient, while strip-cutting enables mechanized, cost-effective management and supports long-term production. Intensive strip-cutting disturbs bamboo ecosystems, altering soil, litter and understory vegetation. This may reduce long-term productivity despite moso bamboo’s rapid growth, especially in the mountainous areas like Anji, Zhejiang. To balance ecological and production goals, we evaluated strip-cutting widths of 3, 5, and 8 m under three slope classes, 5–14° (gentle, SL1), 15–24° (moderate, SL2), and 25–34° (steep, SL3), focusing on bamboo growth recovery and understory vegetation diversity. Compared with uncut control plots, the number of herbaceous and shrub species increased in all treatment plots. In 5 m moderate slope plots, shoot and culm numbers were 27% and 13% higher than those in the 3 m and 8 m plots, and 37% higher than uncut control plots. Herb species diversity, as reflected by the Shannon–Wiener (*H*′), Simpson (*D*), and Margalef richness (*R*) indices, was high in the narrowest clearcut strips under SL1 and SL3. Pielou’s evenness index (*J*) was high in the 3 and 5 m plots under SL2 and SL3. Shrub species diversity, as indicated by *D* and *R*, was high in 5 and 8 m plots under SL2 and SL3. Principal component analysis (PCA) indicated that under SL2, 5 m strip-cutting width with a score of 0.649 outperformed others. These results suggest that 5 m strip-cutting width under SL2 slope optimizes understory vegetation diversity and supports a synergistic outcome of “high shoot emergence–high culm formation” thereby achieving both ecological and production benefits.

## 1. Introduction

Topography plays a crucial role in shaping vegetation distribution and growth patterns [1,2,3]. Variations in the slope gradient can substantially alter environmental conditions, such as light availability, moisture levels, and soil nutrient content [4], thereby influencing plant growth, development, and regeneration. These changes ultimately lead to different vegetation distribution, biodiversity, and productivity [5,6,7]. Although numerous studies have explored the relationship between topographic and geomorphological factors and vegetation dynamics [8,9,10], a unified consensus has not been reached, likely owing to the complexity of these factors, diversity of vegetation types, and influence of other variable disturbances [11]. Consequently, further investigation is required to elucidate how specific topographic conditions affect the growth and biodiversity of vegetation types in localized regions.

Bamboo forests constitute a vital component of the forest resources in China. According to the Ninth National Forest Resources Inventory [12], China possesses approximately 6.4 million ha of bamboo forests, of which moso bamboo accounts for 4.7 million ha (73%). Moso bamboo is a native, large-running bamboo species with monopodial rhizomes in subtropical China, typically reaching 9–17 m in height and about 5–17 cm in culm diameter in managed plantations [13,14]. This fast-growing species features high biomass yield, rapid regeneration, and short rotation periods, making it valuable both economically and ecologically through functions such as carbon sequestration [15,16,17]. Its excellent material properties support wide application in furniture, plywood, bamboo charcoal, and fiber industries, contributing to rural income and regional development. However, declining harvesting intensity, rising labor costs, and shortages have hindered long-term management operations [18,19,20,21]. Traditional selective harvesting methods are labor-intensive and expensive, limiting their feasibility for large-scale operations. Therefore, developing industrialized and cost-effective harvesting strategies coupled with post-harvest tending to restore ecological functions is essential for advancing the bamboo industry [22,23,24,25,26,27,28].

To address these issues, some scholars have explored alternative harvesting approaches, such as strip-cutting, to improve management efficiency. Compared with traditional selective harvesting, strip-cutting offers advantages such as higher efficiency, lower operational costs, and higher mechanization potential, thereby increasing yield and reducing costs [22,24,29,30,31,32,33,34,35]. However, strip-cutting introduces substantial disturbances to bamboo forest ecosystems, as the canopy gaps created can markedly influence the growth and development of moso bamboo and strongly affect understory vegetation. Previous studies on strip-cutting have mainly focused on flat or gently sloping areas, with limited attention to mountainous terrains where most managed moso bamboo forests are actually located. Although earlier findings indicate that moderate disturbance may enhance species diversity in bamboo forests, limited research has evaluated how different strip-cutting widths and slope gradients shape understory biodiversity [27,36,37,38,39,40].

Fundamentally, strip-cutting imposes varying disturbance intensities, and although large canopy openings in moso bamboo forest tend to close relatively quickly, differences in cutting width and recovery time lead to distinct growth patterns and competitive interactions among understory plants. This study aimed to clarify impact of ecological disturbance under different harvesting intensities, providing insights into two key questions: (1) How do variations in strip-cutting width and slope influence recovery of bamboo stands, including shoot emergence and culm formation? (2) What are the effects of varying harvesting intensity and slope on understory plant diversity and what is the optimal combination of cutting width and slope for moso bamboo? Understanding how understory species respond to strip-cutting is crucial, as it not only helps clarify mechanisms driving biodiversity changes but also provides scientific guidance for balancing production needs with ecological objectives in moso bamboo forest management.

## 2. Results

### 2.1. Species Composition and Importance Value of Understory Vegetation Under Different Strip-Cutting Widths and Slope Gradients

**Figure 1:**
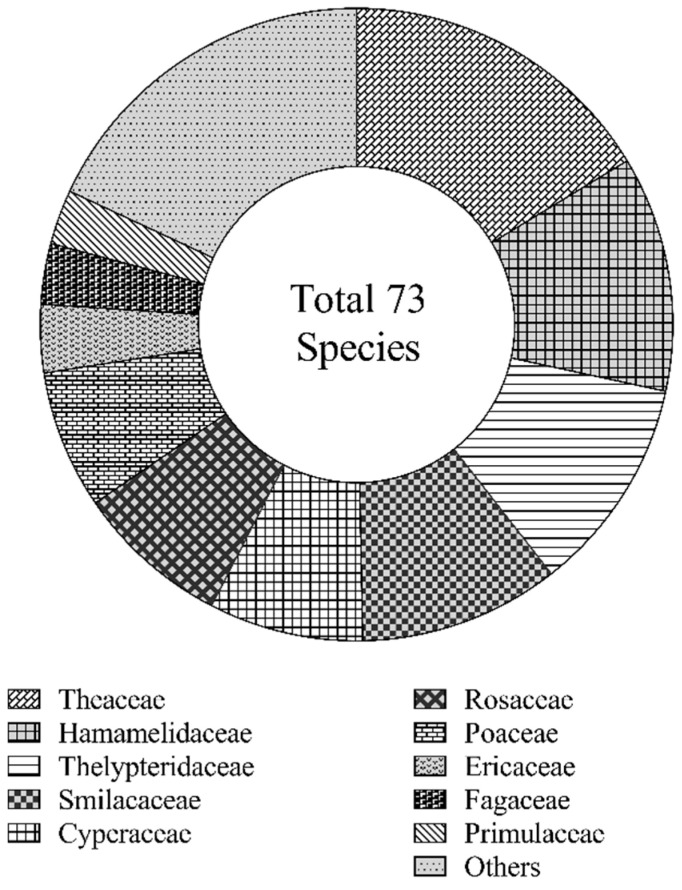
Family composition of understory vegetation.

A total of 73 species belonging to 59 genera and 43 families were identified in the study area. These included 44 shrub species from 33 genera and 28 families, and 29 herbaceous species from 26 genera and 20 families. As shown in Figure 1, the understory vegetation primarily comprised species from Theaceae, Hamamelidaceae, Thelypteridaceae, Smilacaceae, Cyperaceae, Rosaceae, Poaceae, Ericaceae, Fagaceae, and Primulaceae, which collectively accounted for more than 80% of the total recorded species.

**Figure 2:**
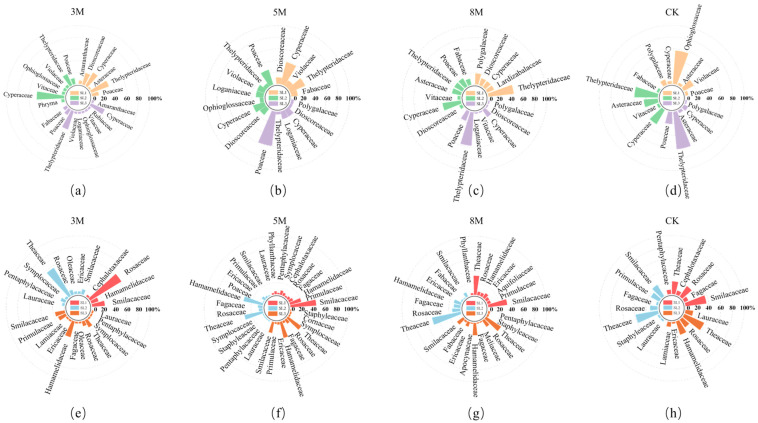
Dominant understory plant species composition under different strip-cutting widths. SL1 (gentle slope), SL2 (moderate slope), and SL3 (steep slope) represent the three slope gradients, whereas (**a**–**d**) and (**e**–**h**) indicate the herbaceous and shrub species family compositions, respectively.

As shown in Figure 2, the composition and number of major understory plant species were higher following strip-cutting than those in CK plots. Across cutting widths of 3, 5, and 8 m, herbaceous species numbers first declined slightly by four species, but later increased by two, representing increases of 181%, 106%, and 144%, respectively, over those in CK plots. For shrubs, species richness showed a minor decline (two species total); however, it still increased by 154%, 142%, and 131%, respectively, relative to that in CK plots. Across slope gradients, herbaceous species increased by one in SL1 (gentle slope), followed by an additional increase of three species in SL2 (moderate) and SL3 (steep), corresponding to increases of 106%, 125%, and 18%, respectively, relative to that in CK plots. Shrub species richness remained unchanged in SL1 and SL2 but increased by four species in SL3, corresponding to increases of 119%, 119%, and 165%, respectively, compared with that in CK plots.

Species importance values (IVs) were calculated for herbaceous and shrub species across plots with varying strip-cutting widths and slope gradients. The species with the highest relative importance were identified and ranked based on their frequency of occurrence across the treatment plots (Table 1). Overall, both species composition and dominance patterns shifted following strip-cutting relative to those in CK plots.

In the herbaceous layer, the dominant species with the highest IVs included *Parathelypteris glanduligera*, *Carex breviculmis*, *Wisteria sinensis*, *Lophatherum gracile*, and *Dioscorea polystachya*. Among these, *P. glanduligera* emerged as dominant under multiple treatments. Under the 3 m cutting width, IVs across slope gradients were 36.3%, 26.3%, and 36.0%, reflecting consistent dominance. In the 5 m cutting-width treatments, IVs fluctuated (46.9%, 14.3%, and 13.7%), indicating that the slope had a stronger influence under medium cutting widths. Under a cutting width of 8 m, IVs subsequently increased to 43.1%, 35.8%, and 43.3%, all of which exceeded the average value (32.8%), indicating strong adaptability to high-intensity disturbance. By contrast, *C. breviculmis* had slightly lower average IVs (29.6%, 26.6%, and 26.3% in the 3, 5, and 8 m plots, respectively) but exhibited smaller variation across slope gradients, demonstrating strong slope adaptability and broad ecological amplitude. In the CK plots, *P. glanduligera* maintained high IVs (46.0% and 52.3%). Conversely, *C. breviculmis* had relatively low IVs in the CK plots (21.2%, 24.0%, and 14.0%, respectively). Plots with a cutting width of 3 m displayed a more concentrated herbaceous composition, with *P. glanduligera* forming strongly dominant communities. In the 5 and 8 m plots, the community structure was more even, with *L. gracile* and *D. polystachya* emerging as co-dominant species alongside *P. glanduligera* and *C. breviculmis*. For example, the IVs of *L*. *gracile* reached 34.1%, 47.6%, and 33.5% in plots 5MSL2, 5MSL3, and 8MSL2, respectively.

In the shrub layer, the species composition was more complex, wherein the dominant species included *Camellia sinensis*, *Camellia japonica*, *Loropetalum chinense*, *Smilax china*, and *Rubus corchorifolius*. *L. chinense* was particularly dominant in 3MSL1 (IV = 47.4%) but declined sharply in other treatments (e.g., IV of 14.1% in 8MSL1). *C. japonica* was highly dominant in both 3MSL3 (37.2%) and 8MSL1 (28.5%). *Fortunearia sinensis* appeared almost exclusively in 3MSL3, with an IV of 35.4%. Members of *Rubus*, particularly *R. corchorifolius*, have demonstrated high regional adaptability and localized dominance. Its IVs were 43.7% and 24.6% in 3MSL1 and CKSL3, respectively. Overall, IVs in the shrub layer fluctuated considerably across the different combinations of cutting widths and slope gradients, with frequent turnover among the dominant species.

Overall, strip-cutting with different combinations of cutting width and slope affected the structure of understory plant communities. Under a cutting width of 3 m, species competition in both the shrub and herbaceous layers was more concentrated, typically around one or two dominant species. By contrast, under cutting widths of 5 and 8 m, species diversity increased, differences among dominant species diminished, and community evenness improved. In the CK plots, the species distribution tended to be more stable, but the dominant species had higher IVs with a trend toward increased competitive exclusion.

### 2.2. Effects of Strip-Cutting Width and Slope Gradient on Shoot Emergence and Culm Growth in Moso Bamboo Forests

The characteristics of shoot emergence and culm development of moso bamboo under different strip-cutting widths and slope gradients are presented in Table 2. Considering the number of shoots, under a cutting width of 3 m, SL3 had the highest shoot density, which was higher than those of SL1 and SL2. Under 5 m, shoot numbers were relatively higher in both SL2 and SL3 but lower in SL1. A cutting width of 8 m resulted in medium-to-high levels of shoot numbers overall, with SL2 and SL3 performing better than SL1. In CK plots, shoot numbers were moderate at all three slopes but were clearly lower than those in combinations, such as a width of 5 m with SL2 and SL3 and a width of 3 m with SL3. New bamboo number showed a similar trend to that of the shoot number, with more stable performance observed under the 5 m cutting width at SL2 and SL3. In both SL2 and SL3, the 5 m width outperformed the 3 and 8 m widths at the same slopes. At 3 m, the number of new bamboos was high in SL3 and moderate in SL1 and SL2. Under the 8 m width, new bamboo numbers were at moderately high levels in SL1 and SL3. Across all slope gradients, the CK treatment had the lowest number of new bamboos. Moderate disturbance (5 m) under SL2 and SL3 conditions was the most conducive to culm formation. Among the strip-cutting treatments, the highest density of bamboo was observed at a width of 5 m with SL3, followed by widths of 3 and 8 m with SL3 and SL2, respectively. At each slope level, the density of bamboo under the 3, 5, and 8 m cutting widths was lower than that in CK plots, but the 5 m width at SL3 achieved a relative balance between maintaining bamboo stock and promoting regeneration. After strip-cutting, DBH generally declined. Among the treatment plots, those with a 3 m width at SL3 maintained relatively high DBH values, exceeding those of most cutting widths on the same slope. In plots with a 5 m width, DBH was lower at SL2 and SL3, whereas those with an 8 m width showed intermediate DBH levels. Canopy closure in the cutting plots was generally lower than or comparable with that of CK plots. Most treatments had canopy closures ranging between 0.65 and 0.76, with those of plots with a 5 m width at SL3 being the lowest. The 8 m plots showed relatively higher canopy closure across all slopes, whereas CK plots maintained high closure in SL2 and SL3.

### 2.3. Impact of Strip-Cutting Width and Slope Gradient on Understory Vegetation Diversity in Moso Bamboo Forests

**Figure 3:**
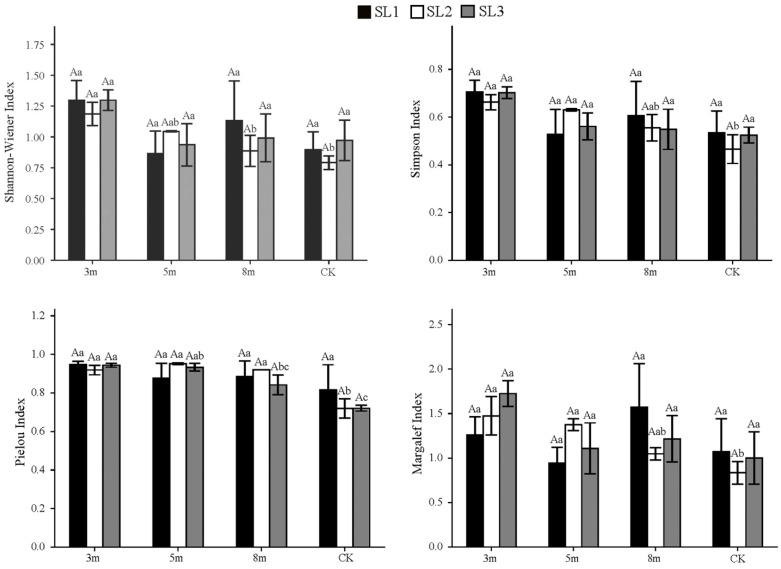
Herbaceous layer diversity indices under different strip-cutting widths and slopes. Different uppercase letters indicate significant differences (*p* < 0.05) among slope gradients within the same strip-cutting width. Different lowercase letters indicate significant differences (*p* < 0.05) among strip-cutting widths within the same slope gradient.

**Figure 4:**
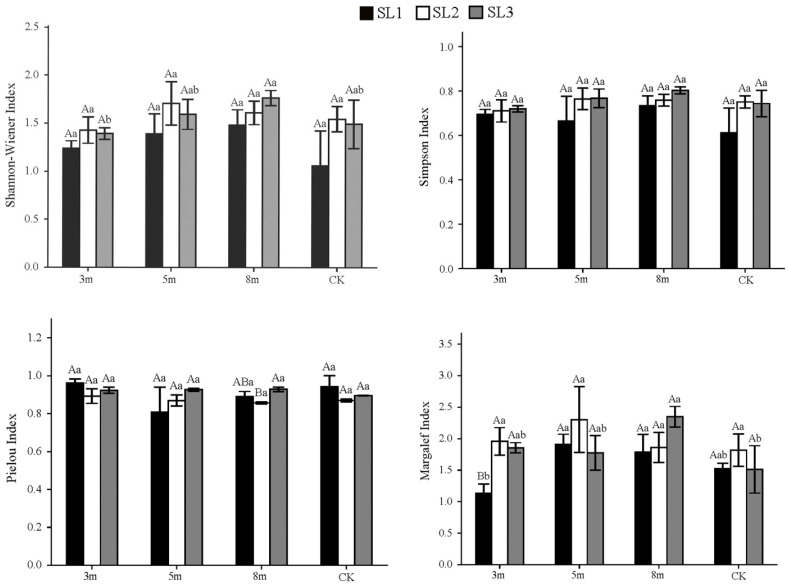
Shrub layer diversity indices under different strip-cutting widths and slopes. Different uppercase letters indicate significant differences (*p* < 0.05) among slope gradients within the same strip-cutting width. Different lowercase letters indicate significant differences (*p* < 0.05) among strip-cutting widths within the same slope gradient.

As shown in Figure 3 and Figure 4, strip-cutting generally increased the understory vegetation diversity indices in moso bamboo forests compared with the CK plots, with only a few exceptions. Under the same cutting width, diversity in the 3 m plots showed a non-linear response to slope: the Shannon–Wiener (*H*′), Simpson (*D*), and Pielou’s evenness indices (*J*) for both the shrub and herbaceous layers first declined and subsequently increased, whereas the Margalef richness index (*R*) increased steadily. However, the differences between the slope levels were not significant. For the herbaceous layer, species diversity under the 3 m width at SL1 and SL3 was consistently higher than those under the 5 m, 8 m, and CK plots. *D* was the highest in 3 m plots across all slopes, whereas CK plots showed the lowest values. *J* values were higher under 3 and 5 m cutting widths at SL2 and SL3, respectively. *R* was also the highest under the 3 m width at SL2 and SL3. Average comparisons across slope gradients further confirmed these patterns: diversity indices for the herbaceous layer were consistently higher under the 3 m cutting width, whereas CK showed the lowest or near-lowest values across all four indices. Considering the slope effects, *H*′ and *D* were slightly higher in SL1 and SL3 than in SL2. *R* was marginally higher in SL3, whereas *J* was similar in SL1 and SL2, both of which exceeded the values observed in SL3.

In contrast to the herbaceous layer, the shrub layer diversity responded more positively to the 5 and 8 m strip-cutting widths across most slope gradients, especially on moderate (SL2) and steep slopes (SL3). Under SL1, *H*′ was the highest in the 8 m plots, followed by the 5 m plots, while 3 m and CK plots had lower *H*′. Under SL2, 5 m plots showed the highest *H*′, followed by 8 m, CK, and 3 m. Under SL3, 5 and 8 m plots had higher *H*′ values than CK and 3 m plots, respectively. *D* followed a similar pattern to *H*′: *D* values in the 8 m plot ranked the highest under SL1 and SL3, while 5 and 8 m plots shared the top rank under SL2. *J* exhibited a slightly different pattern: under SL1, 3 m and CK plots showed higher evenness than 5 and 8 m plots; under SL3, the 5 and 8 m plots shared the highest *J* values, slightly exceeding those of the 3 m and CK plots; and under SL2, the 3 m plot showed a slightly higher *J* value, followed by 5 and 8 m plots, whereas CK had the lowest *J* value. *R* generally favored 5 and 8 m widths: under SL1, 5 m > 8 m > CK > 3 m; SL2, 5 m > 3 m > 8 m > CK; and SL3, 8 m > 3 m > 5 m > CK). Averaged across slopes, *H*′ and *D* ranked 8 m ≥ 5 m > CK ≈ 3 m. Both indices increased with slope steepness, while *R* peaked at SL2 and J at SL3. Overall, 5–8 m strip-cutting widths on moderate and steep slopes best enhanced shrub diversity and richness.

### 2.4. Principal Component Analysis of Diversity Indices, Shoot Emergence, and Culm Growth in Strip-Cutting Moso Bamboo Forests

Based on the competitive relationship between moso bamboo and understory vegetation, principal component analysis (PCA) was conducted after applying min-max normalization to the variables. Positive effects were assigned to indicators related to shoot emergence and culm growth, whereas negative effects were assigned to aborted shoots with different strip-cutting widths and slope gradients. As presented in Table 3, the eigenvalue of the first principal component was 5.485, explaining 39.176% of the variance; the second principal component had an eigenvalue of 4.224, explaining 30.172% of the variance; and the third principal component had an eigenvalue of 2.078, contributing to 14.842%. Overall, the first three principal components accounted for 84.191% of the total variance, indicating that they captured nearly all the information in the original dataset (cumulative variance explained ≥80%). The first principal component was mainly characterized by high loadings for the herbaceous layer *H*′ > DBH > *R* of the herbaceous layer > *J* of the herbaceous layer > *J* of the shrub layer > *D* of the shrub layer, primarily reflecting bamboo growth and herbaceous vegetation characteristics. The second principal component showed high loadings for the number of new bamboos > number of shoots > density > *J* of the shrub layer > *H*′ of shrub layer, mainly representing bamboo shoot emergence and growth characteristics. The third principal component had high loadings for canopy closure > *R* of the herbaceous layer > *R* of the shrub layer > *H*′ of the herbaceous layer, mainly representing understory bamboo growth and shrub layer vegetation characteristics in moso bamboo forests.

Based on the results of PCA (Table 3), a set of 14 variables was selected to comprehensively evaluate shoot emergence, culm growth, and understory vegetation diversity in moso bamboo forests under different strip-cutting widths and slope gradients. These indicators included numbers of shoots, aborted shoots, and new bamboos; density; canopy closure; *H’*, *D*, *J*, and *R* of the shrub layer; and the corresponding four indices for the herbaceous layer. To consider the differing contributions of each variable, weighted summation and membership function values were calculated. Using the evaluation equation $Z=\Sigma W_{i}\times X_{\left(U\right)}$, the comprehensive effect scores were computed for the nine treatment combinations (Table 4).

## 3. Discussion

### 3.1. Responses of Understory Vegetation Composition and Diversity to Different Strip-Cutting Width and Slope Gradient

In this study, we found that the study site exhibited relatively high species richness, with a balanced composition of species across families. Although strip-cutting introduces anthropogenic disturbance, it can enhance understory species richness and compositional diversity under specific combinations of cutting width and slope. Patterns in species composition and importance values showed clear species-specific responses to altered microenvironments. *P. glanduligera*, which had the high IVs in CK plots, exhibited strong competitive ability under undisturbed canopies, whereas *C. breviculmis* maintained relatively stable IVs in moderately disturbed conditions, indicating its suitability for partially opened canopies. *L. gracile* responded positively to wider canopy gaps, while *L. chinense* favored narrower strip-cutting widths on gentle slopes, likely to reflect an interaction between moderate light levels and stable microclimatic conditions. *C. japonica* dominated both 3MSL3 and 8MSL1, reflecting its adaptability to both small-width/steep-slope and large-width/low-slope environments. *F. sinensis* exhibited niche specialization associated with slope or microtopographic settings. Members of *Rubus*, particularly *R. corchorifolius*, had competitive success in slightly disturbed environments. Patterns in understory diversity indices further indicated that narrower strip-cutting widths promoted more balanced coexistence within the herbaceous layer. Moderate light availability and soil disturbance created by smaller cutting widths facilitated the rapid growth and coexistence of herbaceous species. By contrast, a larger cut width or the absence of harvesting may lead to dominance by a few competitive species or cause drier and bitter microenvironments, thereby suppressing overall diversity and evenness. In the shrub layer, diversity increased mainly at SL2 and SL3, although the strong dominance of several species in these conditions led to a slight reduction in evenness. 5–8 m strip widths on moderate and steep slopes best enhanced shrub diversity and richness. The composition and diversity of vegetation are influenced by both the cutting width and slope gradient [41]. Lorenzo et al. [42] and Zhang et al. [43] reported a significant correlation between the plant species diversity and slope. Slope is the second most influential topographic factor after elevation [44], and in some studies, it is considered the primary factor influencing understory vegetation [45]. At finer spatial scales, variables such as the slope gradient, aspect, and elevation are key environmental constraints affecting the understory plant diversity [46].

### 3.2. Effects of Different Strip-Cutting Width and Slope Gradient on Moso Bamboo Regeneration

Shoot emergence and culm growth further reflected the combined influence of canopy structure and slope. Increased slope combined with moderate cutting width substantially promoted shoot emergence, and the highest culm formation occurred under 5 m strips at SL2 and SL3, implying that moderate disturbance optimizes light availability and improves post-disturbance redistribution of water and nutrients. The number of standing culms was higher in the CK treatment, showing a stock advantage in unharvested bamboo stands. Moderate strip widths on steeper slopes also helped support new bamboo growth. Diameter at breast height (DBH) was the highest in CK plots at SL1 and SL3, indicating that long-term non-harvested stands tended to develop greater culm diameter. Canopy closure decreased noticeably in strip-cutting plots, confirming that harvesting opened the canopy and improved understory light and thermal conditions. When considered alongside shoot and culm dynamics, these results suggest that moderately reducing canopy closure can promote shoot emergence and culm formation, whereas excessive opening may lead to reduced DBH, increased soil dryness, and heat stress. In this study, strip-cut moso bamboo served as the dominant overstory species, whereas the understory vegetation served as a competing plant community. PCA revealed that bamboo shoot emergence and culm growth were generally negatively correlated with the shrub layer diversity but positively correlated with the herbaceous layer diversity. This may be because shrubs compete more with moso bamboo for soil nutrients compared to herbaceous and vine species. Additionally, the ground cover provided by herbaceous and vine plants may reduce surface evaporation, helping conserve soil moisture [11,46,47].

### 3.3. Ecological and Management Implications of Strip-Cutting in Moso Bamboo Forests

Cutting width and slope are key factors that alter the habitat conditions for vegetation, such as light availability, soil moisture, and nutrient dynamics, which subsequently affect the growth, development, and regeneration of plant communities [6,7,8,48]. In this study, the 5 m strip-cutting width, which was applied on moderate slopes (SL2, 15–24°), resulted in favorable shoot emergence and culm formation in moso bamboo, while also maintaining a relatively high density of standing culms. This performance may be attributed to the superior soil conditions commonly found on moderate slopes, including greater soil depth, moisture retention, and nutrient availability [49]. 5 m strip-cutting width creates balanced canopy openings that enhance light availability without causing excessive soil drying, thereby supporting shoot emergence. This intermediate light environment is sufficient to stimulate the emergence of shoots while still preserving adequate shade to prevent excessive evapotranspiration and thermal stress. In addition, nutrient redistribution and retention after strip-cutting are more effective on moderate slopes, which reduces resource limitation for emerging shoots. These conditions decrease intraspecific competition and promote higher culm formation. Moderate slopes provide optimal soil water dynamics compared with gentle or steep slopes. On gentle slopes, water may accumulate and reduce aeration, limiting root development, whereas steep slopes promote rapid runoff and poor infiltration. Moderate slopes strike a balance by enhancing infiltration while retaining sufficient moisture, thereby supporting both shoot emergence and culm formation. Post-harvest redistribution of organic matter and nutrients is more effectively retained on moderate slopes. Unlike steep slopes, where nutrients are easily lost through erosion, or gentle slopes, where nutrient accumulation may lead to localized resource imbalances, moderate slopes facilitate relatively even nutrient availability, reducing competition among culms for essential resources. 5 m strip-cutting width provides enough space for root expansion and lateral rhizome growth while avoiding the excessive root exposure or instability associated with larger openings. These findings corroborate those of Huang et al. [50], Wu et al. [51], Xiao et al. [52], Ziting et al. [53], and Guancheng et al. [11]. The CK plots exhibited the lowest or near-lowest values for herbaceous diversity indices, and the shrub layer diversity was also frequently lower in CK plots, suggesting that harvesting disturbances can positively restructure understory vegetation communities.

Traditional selective harvesting requires substantial manual labor and has become increasingly difficult to maintain as labor costs rise and productivity declines in poorly managed bamboo forests. In contrast, strip-cutting enables more concentrated and efficient harvesting operations, facilitates future mechanization, and can reduce operational costs, offering a practical pathway for maintaining the economic viability of bamboo-based industries. Moderate strip-cutting width and slope not only promote shoot regeneration and improve culm formation but also create heterogeneous light and soil environments that support higher understory species richness, contributing to biodiversity conservation within managed bamboo forests. Our identification of an optimal strip-cutting width and slope provides practical guidance for preventing over-harvesting and preserving understory diversity, thereby mitigating the risks of soil erosion, nutrient loss, and the degradation of water conservation functions. These findings illustrate how strip-cutting strategies can effectively balance productivity and ecological preservation, providing important insights for management of bamboo forests. The potential effects of alternative cutting-width ranges, different slope classifications, or varying survey periods were not investigated in this study. Furthermore, limitations regarding the relatively short post-harvest observation period and the limited number of experimental replications exist. Future studies should address these aspects to capture longer-term recovery dynamics and improve the generalizability of the findings.

## 4. Materials and Methods

### 4.1. Study Area and Experimental Design

**Figure 5:**
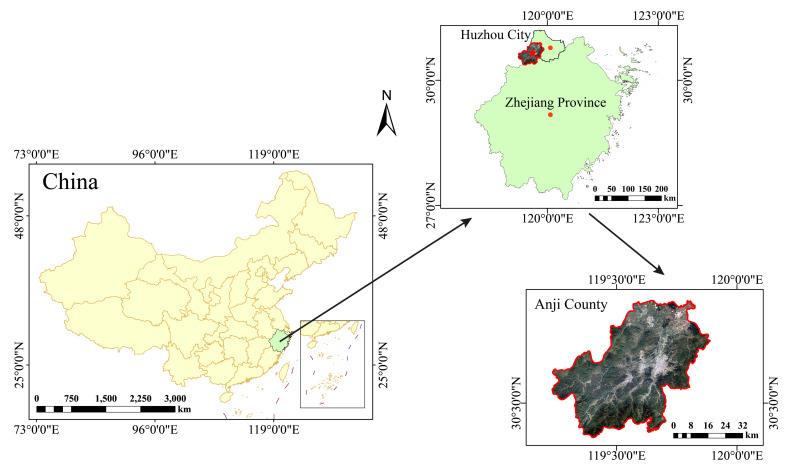
Location of the study area.

The study area is located in Anji County, Huzhou City, Zhejiang Province (Figure 5), encompassing a total area of 1886 km^2^ and predominantly characterized by mountainous and hilly terrain. Situated in the northwestern part of Zhejiang (30° 23′–30° 53′ N, 119° 14′–119° 53′ E), this region experiences a subtropical maritime monsoon climate with mild year-round temperatures, coincident rainfall and temperature seasonality, abundant precipitation, and distinct seasonal variations. The long-term average annual temperature is 16.5 °C, and the mean annual precipitation is 1478 mm. Forests cover approximately 69.4% of the county’s area, with 132,000 ha of forested mountains, including 63,300 ha of bamboo forests. Moso bamboo stands occupy 49,900 ha, accounting for 37.8% of the total forest area. These forests are predominantly managed using traditional selective manual harvesting methods. The main soil types in Zhejiang Province are red soil and yellow soil, with a pH ranging from 4.5 to 5.2 and a soil moisture content of 15% to 35%. The ages of the moso bamboo stands in the cutting strips are all I-degree (≤1 year), whereas those in the reserve strips and CK include II to IV-degree (approximately 2–8 years old or older). The main understory shrub species in the study area include *S. china*, *L. chinense*, *C. sinensis*, and *C. japonica*. The dominant herbaceous species include *P. glanduligera*, *C. breviculmis*, *L. gracile*, and *D. polystachya*.

**Figure 6:**
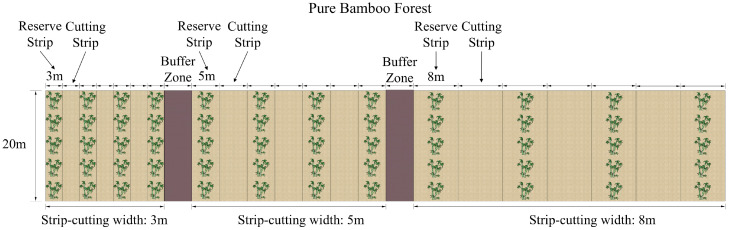
Schematic diagram of establishment of strip-cutting plots.

This study focused on monospecific stands of moso bamboo subjected to strip-cutting. Moso bamboo exhibit a characteristic on- and off-year cycle, in which years of abundant shoot production (“on-year”) alternate with years of low shoot emergence (“off-year”). In November 2023 (off-year), typical pure moso bamboo stands were selected for strip-cutting treatments, with a 8 m buffer zone established between plots subjected to different cutting widths (Figure 6). Strip-cutting removed all bamboo culms within the plot, while two adjacent reserve plots were maintained to provide physiological support and nutrient supply to the cutting plot. No management practices were carried out in all of the sample plots during the restoration period. The harvesting operations were completed in December 2023. From April to June 2024 (on-year), newly emerged shoots were surveyed, and understory vegetation diversity was assessed in August 2024.

Based on the spatial extent of the influence of the underground rhizome system of moso bamboo on shoot development and forest recovery [54], nine strip-cutting treatment plots were established each with three replications, incorporating a factorial combination of three cutting widths and three slope classes (Table 5). The cutting widths were set at 3, 5, and 8 m with a fixed cutting length of 20 m for all treatments. The slope gradients were categorized as follows: SL1, gentle slope (5–14°); SL2, moderate slope (15–24°); and SL3, steep slope (25–34°). Reserve strips of equal width to the cutting strips were established between adjacent cutting areas, and each treatment combination was replicated thrice. Trenches were excavated around the sample plots for isolation. The trenches were 50 cm in depth. Rhizomes were cut off, which could effectively prevent the long-distance transportation of nutrients from the bamboo outside the study area. Additionally, for each slope class, three 20 m × 20 m plots were designated as the uncut control (CK) plots. Protective fencing and signage were installed around the study area to ensure the protection of all experimental plots.

### 4.2. Data Collection and Index Calculation

From April to October 2024, field measurements were conducted in all the treatment and control plots to assess moso bamboo growth within the strip-cutting areas. The measured variables included the DBH, number of emerging shoots, new bamboo culms, and standing culms. In September 2024, a 180° fisheye lens and model D70 digital camera (Nikon, Tokyo, Japan) were used to capture images from below the bamboo canopy. The images were loaded into canopy analysis software (HemiView 2.1 SR4, Delta-T Devices Ltd., Burwell, Cambridge, UK) for canopy closure analysis. In August, the understory vegetation was rigorously surveyed. In each plot, three shrub quadrats (3 m × 3 m) were established. Within each shrub quadrat, three standard herbaceous subplots (1 m × 1 m) were randomly established. The recorded parameters included the species composition, canopy cover, and abundance. The IV was calculated as follows.

For shrub and herbaceous layers,

(1) $$IV=\frac{\left(Relative\;Abundance+Relative\;Coverage+Relative\;\mathrm{Frequency}\right)}{3}$$

The understory species diversity in moso bamboo forests was assessed using IV as the basis for calculating α-diversity indices. The α-diversity indices were calculated using the following equations:

Shannon–Wiener diversity index (*H*′):

(2) $$H^{\prime }=-\sum_{i=i}^{S}P_{i}lnP_{i}$$

Simpson diversity index (*D*):

(3) $$D=1-\sum_{i=1}^{S}P_{i}^{2}$$

Pielou’s evenness index (*J*):

(4) $$J=\frac{-\sum P_{i}lnP_{i}}{lnS}$$

Margalef richness index (*R*):

(5) $$R=\frac{S-1}{ln(N)}$$

where *S* is the total number of species within the plot; and $P_{i}$ is the relative abundance of species *i*, defined as $P_{i}=\frac{n_{i}}{N}$, where $n_{i}$ is the number of individuals of species *i* and *N* is the total number of individuals of all species in the plot.

### 4.3. Data Analysis

Data management was conducted using Excel 2016, and graphical visualizations were produced using Origin 2022 and GraphPad Prism 9.5.1. Python 3.11.3 was used for statistical analyses, including analysis of variance and post hoc multiple comparisons through the least significant difference test (statistical significance set at *p* < 0.05). Normality and homogeneity of variance assumptions were verified using Shapiro–Wilk and Levene’s tests, respectively. PCA was conducted using R 4.5.0.

## 5. Conclusions

In this study, we demonstrate that strip-cutting can effectively enhance the species richness and diversity of understory vegetation in moso bamboo forests. Comprehensive analysis suggests that a 5 m cutting width on moderate slopes (15–24°) is optimal; it not only facilitates vigorous shoot emergence and culm formation in moso bamboo but also supports a favorable level of understory vegetation growth and diversity. By identifying an optimal combination of strip-cutting width and slope, this study provides practical guidance for harvesting intensity and scientific basis for long-term management of bamboo forests that integrates ecological conservation with production efficiency. Appropriate strip-cutting designs can not only promote effective post-harvest recovery of bamboo stands but also help maintain understory plant diversity, thereby reducing the risks of excessive disturbance. These practices may mitigate potential soil erosion, nutrient loss, and the degradation of water conservation functions commonly associated with over-harvesting. Furthermore, after shoot emergence, proper management measures should be adopted to minimize shoot abortion. These may include soil mounding around the base of bamboo culms on slopes and appropriate thinning of surplus shoots. Moreover, the selective removal of shrub vegetation is recommended to reduce competition for soil nutrients while also retaining herbaceous and vine species to help conserve soil moisture by reducing surface evaporation.

## Figures and Tables

**Table 1:** Importance values of dominant herbaceous and shrub species under different strip-cutting widths and slopes.

	Species	Importance Values/%												
		3M SL1	3M SL2	3M SL3	5M SL1	5M SL2	5M SL3	8M SL1	8M SL2	8M SL3	Average	CK SL1	CK SL2	CK SL3
Herbaceous layer	*Parathelypteris glanduligera*	36.3	26.3	36.0	46.9	14.3	13.7	43.1	35.8	43.3	32.8	-	46.0	52.3
	*Wisteria sinensis*	-	-	26.4	36.6	-	-	-	33.6	-	32.2	-	37.1	-
	*Lophatherum gracile*	21.9	17.6	13.9	-	34.1	47.6	-	33.5	31.2	28.5	33.0	-	22.8
	*Dioscorea polystachya*	23.7	-	-	57.0	52.3	11.5	22.0	19.8	12.5	28.4	-	-	-
	*Gardneria multiflora*	-	-	12.4	-	39.5	46.7	-	-	11.9	27.6	-	-	-
	*Carex breviculmis*	24.3	39.5	24.9	26.5	24.7	28.5	28.9	29.3	20.6	27.5	21.2	24.0	14.0
	*Syneilesis aconitifolia*	-	-	-	-	-	-	-	26.6	-	26.6	-	46.6	13.3
	*Sceptridium ternatum*	-	30.0	14.5	-	20.0	-	-	-	-	21.5	57.6	-	-
Shrub layer	*Camellia sinensis*	-	31.7	18.1	-	26.1	25.1	35.1	31.1	16.8	26.3	-	17.9	30.8
	*Loropetalum chinense*	47.4	-	20.0	13.6	29.5	26.2	14.1	14.5	25.3	23.8	-	-	7.0
	*Camellia japonica*	-	30.1	37.2	-	10.6	20.9	28.5	13.9	19.9	23.0	21.0	34.0	11.9
	*Smilax china*	26.3	12.7	21.9	33.3	9.1	19.5	23.2	10.1	11.8	18.7	34.4	16.4	-
	*Rubus corchorifolius*	43.7	15.7	12.4	-	-	7.4	9.8	15.6	7.7	16.0	-	14.1	24.6
	*Quercus* × *leana*	-	-	10.0	12.1	13.5	17.1	-	17.2	9.9	13.3	57.5	12.5	-
	*Rhododendron simsii*	-	15.0	18.0	-	9.1	9.6	13.5	6.5	18.7	12.9	-	-	6.3
	*Ardisia japonica*	-	-	20.1	14.5	6.4	7.2	12.0	-	-	12.0	-	21.3	-

**Table 2:** Post-harvest shoot emergence and growth of moso bamboo in response to different strip-cutting widths and slope gradients.

Factor		Shoots (Culms·ha^−1^)	New Bamboo (Culms·ha^−1^)	Density (Culms·ha^−1^)	DBH (cm)	Canopy Closure
Width	Slope					
3M	SL1	3888.89 ± 433.90 Aa	2111.11 ± 111.11 Aa	2111.11 ± 277.78 Ab	7.76 ± 0.26 Bb	0.71 ± 0.01 ABab
	SL2	3222.22 ± 529.97 Aa	2333.33 ± 509.18 Aa	2333.33 ± 419.44 Ab	7.04 ± 0.22 Cab	0.68 ± 0.02 Bb
	SL3	4300.00 ± 409.61 Aa	2666.67 ± 252.76 Aab	2666.67 ± 197.20 Abc	8.51 ± 0.22 Ab	0.75 ± 0.02 Aa
5M	SL1	2366.67 ± 176.38 Bb	1633.33 ± 260.34 Ba	1633.33 ± 202.76 Bb	7.81 ± 0.25 Ab	0.70 ± 0.01 Bab
	SL2	4100.00 ± 1137.25 ABa	3233.33 ± 864.74 ABa	2600.00 ± 702.38 ABb	6.67 ± 0.18 Bb	0.75 ± 0.02 Aa
	SL3	4320.00 ± 241.66 Aa	3380.00 ± 231.08 Aa	3400.00 ± 219.09 Ab	7.13 ± 0.16 Bc	0.65 ± 0.01 Cb
8M	SL1	3208.33 ± 342.96 Aab	2458.33 ± 433.51 Aa	1875.00 ± 72.17 Bb	7.91 ± 0.18 Ab	0.75 ± 0.00 Aa
	SL2	3604.17 ± 240.26 Aa	2875.00 ± 165.36 Aa	2645.83 ± 126.72 Ab	6.76 ± 0.18 Bb	0.74 ± 0.00 Aab
	SL3	3412.50 ± 232.68 Aab	2725.00 ± 264.13 Aab	2350.00 ± 235.19 ABc	7.22 ± 0.14 Bc	0.76 ± 0.01 Aa
CK	SL1	2558.33 ± 96.10 Ab	1566.67 ± 230.19 Aa	4558.33 ± 282.97 Aa	9.31 ± 0.23 Aa	0.70 ± 0.03 Ab
	SL2	3000.00 ± 350.30 Aa	2166.67 ± 289.16 Aa	4291.67 ± 210.32 Aa	7.72 ± 0.27 Ba	0.74 ± 0.02 Aa
	SL3	2941.67 ± 572.70 Ab	2050.00 ± 355.61 Ab	4708.33 ± 729.20 Aa	9.72 ± 0.19 Aa	0.74 ± 0.02 Aa

Note: Different uppercase letters indicate significant differences (*p* < 0.05) among the slope gradients within the same strip-cutting width. Different lowercase letters indicate significant differences (*p* < 0.05) among the strip-cutting widths within the same slope gradient.

**Table 3:** Principal component matrix and weight of each factor.

Variable	Principal Component			Weight
	1	2	3	
DBH (cm)	0.576	−0.412	0.322	0.053
Shoots (Culms·ha^−1^)	0.346	0.910	−0.086	0.080
Aborted shoots (Culms·ha^−1^)	−0.928	−0.164	0.083	0.093
New bamboo (Culms·ha^−1^)	−0.298	0.933	−0.038	0.080
Density (Culms·ha^−1^)	−0.101	0.879	−0.359	0.069
Canopy closure	−0.051	0.051	0.880	0.031
Shrub layer Shannon–Wiener Diversity Index: *H’*	−0.816	0.507	0.258	0.093
Shrub layer Simpson Diversity Index: *D*	0.531	−0.747	−0.262	0.077
Shrub layer Pielou’s Evenness Index: *J*	0.554	0.546	0.062	0.057
Shrub layer Margalef Richness Index: *R*	−0.725	0.200	0.476	0.067
Herbaceous layer Shannon–Wiener Diversity Index: *H’*	0.904	0.151	0.323	0.092
Herbaceous layer Simpson Diversity Index: *D*	−0.904	−0.233	−0.172	0.091
Herbaceous layer Pielou’s Evenness Index: *J*	0.572	0.489	−0.399	0.060
Herbaceous layer Margalef Richness Index: *R*	0.573	0.287	0.630	0.057
Eigenvalue	5.485	4.224	2.078	
Proportion of Variance/%	39.176	30.172	14.842	
Cumulative Variance Explained/%	39.176	69.348	84.191	

**Table 4:** Comprehensive score of shoot and bamboo growth and understory diversity characteristics of moso bamboo.

Plot	Full-Factor Weighted Scoring	
	Score	Rank
5MSL2	0.649	1
5MSL3	0.626	2
3MSL3	0.624	3
8MSL3	0.596	4
8MSL1	0.561	5
8MSL2	0.544	6
3MSL2	0.520	7
3MSL1	0.440	8
5MSL1	0.398	9

**Table 5:** Basic information of the sample plots.

Plot	Longitude	Latitude	Area/m^2^	Slope/(°)	Altitude/m	Aspect	Density/(stems∙hm^−2^)	Average DBH/cm
3MSL1	119°30′3″ E	30°29′31″ N	60	14	466	SW	2111	7.76
5MSL1	119°30′2″ E	30°29′31″ N	100	13	470	S	1633	7.81
8MSL1	119°30′1″ E	30°29′31″ N	160	13	470	S	1875	7.91
3MSL2	119°30′3″ E	30°29′34″ N	60	24	500	SE	2333	7.04
5MSL2	119°30′1″ E	30°29′35″ N	100	23	472	S	2600	6.67
8MSL2	119°29′43″ E	30°29′38″ N	160	23	395	SE	2645	6.76
3MSL3	119°29′59″ E	30°29′38″ N	60	31	504	S	2666	7.91
5MSL3	119°30′0″ E	30°29′35″ N	100	33	503	SW	3400	6.76
8MSL3	119°29′58″ E	30°29′36″ N	160	33	505	SE	2350	7.22
CKSL1	119°30′3″ E	30°29′30″ N	400	13	466	SE	4558	9.31
CKSL2	119°29′42″ E	30°29′39″ N	400	22	399	SW	4291	7.72
CKSL3	119°29′58″ E	30°29′35″ N	400	30	475	SW	4708	9.72

Note: 3M, 5M, and 8M represent the width of the cutting strips, whereas SL1, SL2, and SL3 denote gentle, moderate, and steep slopes, respectively; CK represents the uncut control plots with traditional manual selective harvesting. SL1, SL2, and SL3 after “CK” indicate gentle, moderate, and steep slopes, respectively.

## Data Availability

Data are contained within the article.
